# First molecular detection and characterization of zoonotic *Bartonella* species in fleas infesting domestic animals in Tunisia

**DOI:** 10.1186/s13071-017-2372-5

**Published:** 2017-09-19

**Authors:** Saba Zouari, Fatma Khrouf, Youmna M’ghirbi, Ali Bouattour

**Affiliations:** Université de Tunis El Manar, Institut Pasteur de Tunis, Laboratoire d’Epidémiologie et de Microbiologie Vétérinaire LR11IPT03, Service d’Entomologie Médicale, 1002 Tunis-Belvédère, Tunisie

**Keywords:** *Bartonella elizabethae*, *B. henselae*, *B. clarridgeiae*, *Ctenocephalides felis*, *C. canis*, *Pulex irritans*, Bartonellosis, Zoonotic *Bartonella* species, Tunisia

## Abstract

**Background:**

Bartonellosis is an emerging vector-borne disease caused by different intracellular bacteria of the genus *Bartonella* (Rhizobiales: *Bartonellaceae*) that is transmitted primarily by blood-sucking arthropods such as sandflies, ticks and fleas*.* In Tunisia, there are no data available identifying the vectors of *Bartonella* spp. In our research, we used molecular methods to detect and characterize *Bartonella* species circulating in fleas collected from domestic animals in several of the country’s bioclimatic areas.

**Results:**

A total of 2178 fleas were collected from 5 cats, 27 dogs, 34 sheep, and 41 goats at 22 sites located in Tunisia’s five bioclimatic zones. The fleas were identified as: 1803 *Ctenocephalides felis* (83%) (Siphonaptera: Pulicidae), 266 *C. canis* (12%) and 109 *Pulex irritans* (5%) (Siphonaptera: Pulicidae). Using conventional PCR, we screened the fleas for the presence of *Bartonella* spp., targeting the citrate synthase gene (*gltA*)*. Bartonella* DNA was detected in 14% (121/866) of the tested flea pools [estimated infection rate (EIR) per 2 specimens: 0.072, 95% confidence interval (CI): 0.060–0.086]. The *Bartonella* infection rate per pool was broken down as follows: 55% (65/118; EIR per 2 specimens: 0.329, 95% CI: 0.262–0.402) in *C. canis*; 23.5% (8/34; EIR per 2 specimens: 0.125, 95% CI: 0.055–0.233) in *P. irritans* and 6.7% (48/714; EIR per 2 specimens: 0.032, 95% CI: 0.025–0.045) in *C. felis.* Infection rates, which varied significantly by bioclimatic zone (*P* < 0.0001), were highest in the humid areas. By sequencing, targeting the *gltA* gene and the 16S–23S rRNA Intergenic Spacer Regions (ITS), we identified three *Bartonella* zoonotic species: *B. elizabethae*, *B. henselae*, *B. clarridgeiae*, as well as uncharacterized *Bartonella* genotypes.

**Conclusions:**

To the best of our knowledge, this is the first time that fleas in Tunisia have been shown to carry zoonotic species of *Bartonella*. The dog flea, *Ctenocephalides canis*, should be considered the main potential vector of *Bartonella*. Our study not only provides new information about this vector, but also offers a public health update: medical practitioners and farmers in Tunisia should be apprised of the presence of *Bartonella* in fleas and implement preventive measures.

**Electronic supplementary material:**

The online version of this article (10.1186/s13071-017-2372-5) contains supplementary material, which is available to authorized users.

## Background

Global changes, including climate and land use, are among the most significant influences on the distribution and density of hematophagous arthropod species, which may affect zoonotic diseases including bartonellosis, caused by bacteria of the genus *Bartonella* (fastidious hemotropic Gram-negative, facultative intracellular organisms) belonging to the α2-subgroup of the proteobacteria. These organisms can infect animal and human erythrocytes and endothelial cells and cause polymorphic clinical diseases [1]. A host can be infected by *Bartonella* by transfusions/organ transplants, arthropod saliva (fleas, sand flies, lice and ticks) or by flea feces in the case of Cat-Scratch-Disease (CSD) [1, 2].

Several tools that are useful and more sensitive than culture and serological techniques, including conventional PCR, RFLP, MST, MLVA and also the pre-enrichment culture using *Bartonella*-Alpha-Proteobacteria-Growth Medium combined with PCR and sequencing (ePCR) [3], have been developed to simultaneously detect and characterize *Bartonella* species and subspecies in hosts and vectors [4, 5]. More than 35 species and three subspecies have been identified to date, of which 17 species are involved or associated with an expanding spectrum of animal and human diseases. These include Carrion’s disease, trench fever, bacillary angiomatosis, endocarditis and CSD [6, 7]. CSD the most common zoonotic infection caused by *Bartonella henselae*, which is transmitted by cat fleas, *Ctenocephalides felis. Bartonella henselae*, which is frequently transmitted to humans by a cutaneous trauma (scratch and/or bite) from an infected cat [8], was recently implicated in an intraocular inflammation caused by CSD in Tunisia [9]. Several cases of *B. quintana* endocarditis were also reported [10, 11].

Serological studies conducted in Tunisia showed canine seropositivity for *B. vinsonii*, *B. henselae*, *B. clarridgeiae* and *B. bovis*. This correlated well with tick and/or flea infestations of the dogs that were tested [12]. Moreover, a recent molecular study detected “*Candidatus* B. merieuxii” DNA in dogs [13]. While several clinical, molecular and serological studies have demonstrated that *Bartonella* species circulate among patients and dogs in Tunisia [8–10, 12], no data about the role of fleas as potential vectors are available.

This research used molecular methods to detect and characterize *Bartonella* species circulating in fleas collected from domestic animals and to compare its infection rates in Tunisia’s various bioclimatic areas.

## Methods

### Flea collection and identification

**Fig. 1:**
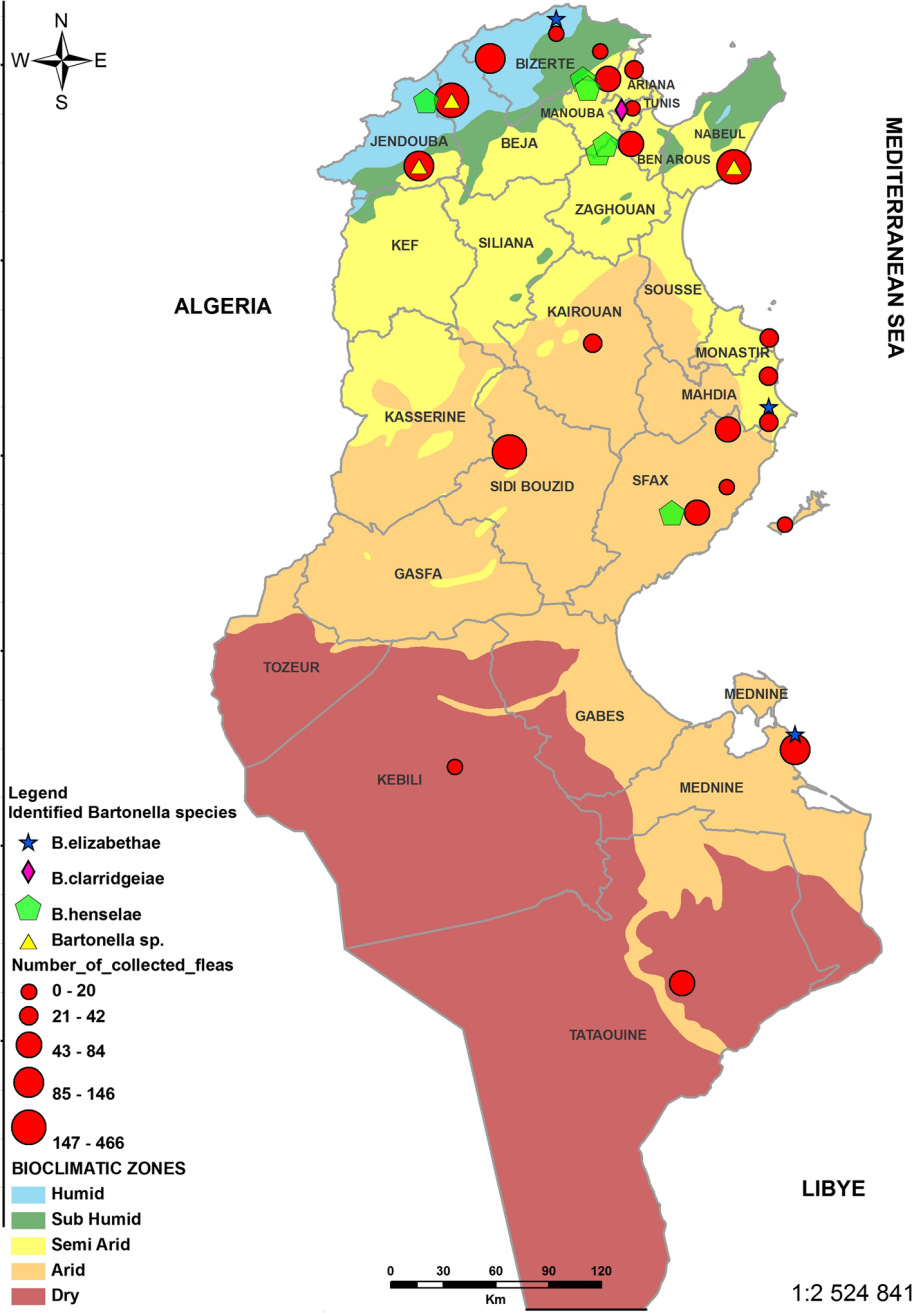
Map of Tunisia showing the distribution of flea collection sites and repartition of *Bartonella* infected fleas according to bioclimatic areas

An entomological investigation was carried out to collect fleas infesting domestic animals (dogs, cats, goats and sheep) in 22 sites located in Tunisia’s five bioclimatic zones (humid, sub-humid, semi-arid, arid and Saharan, Fig. 1). Individual fleas were placed in labeled flasks and stored in 70% ethanol. All specimens were identified to species level using the standard taxonomic keys [14]. A distribution map of collected fleas, according to the bioclimatic areas, was made using ArcGIS 9 software (version 9.3.1).

### DNA extraction from fleas

For DNA extraction, fleas from the same species, of the same sex, from the same host in the same site were pooled (2 fleas per pool, to reduce testing costs). The identified fleas were removed from the 70% ethanol flasks, washed, dried, and minced with disposable scalpels, and placed in 200 μl ATL buffer. We followed the manufacturer’s instructions for the QIAamp® DNA tissue extraction kit (Qiagen, Hilden, Germany). The final elution step was done using 90 μl of AE buffer and then stored at -20 °C for further use. To monitor for contamination during the processes, an extraction control (distilled water) for every 10 flea pools was added in each extraction experiment. The DNA concentration was determined with a NanoDrop spectrophotometer (NanoDrop ND-1000 Technologies, Wilmington, DE), and ranged between 50 and 400 ng/μl. For the PCR reaction, the extracted DNA was diluted to reach a suitable concentration (50 ng/μl).

### Detection of *Bartonella* spp. by PCR, DNA sequencing and phylogenetic relationships

Fleas were screened for the presence of *Bartonella* spp. using the citrate synthase gene (*gltA*). The amplification of *Bartonella* DNA was carried out as described by Norman et al. [15] using the forward primer BhCS.7p (5′-GGG GAC CAG CTC ATG GTG G-3′) and the reverse primer BhCS.1137n (5′-AAT GCA AAA AGA ACA GTA AAC A-3′), yielding a fragment of approximately 379 bp.

PCR amplifications were performed in a final volume of 25 μl containing 2.5 μl of 10× Taq buffer (Invitrogen, CA, USA), 1,5 μl of MgCl_2_ (1.5 mM), 2.5 μl of deoxyribonucleotide triphosphate (200 μM), 0.5 μl of each primer (200 nM) and 0.75 U of Taq Platinum polymerase (Invitrogen, CA, USA), followed by the addition of 3 μl of flea DNA extracts. The amplification reaction was carried out in a thermocycler (Perkin Elmer 2400). PCR cycling conditions were 10 min at 94 °C for an initial denaturation, followed by 35 cycles of 30 s at 94 °C, 45 s at 51 °C, and 30 s at 72 °C, with a final extension of 7 min at 72 °C. Positive and negative controls were included in each series of experiments. To avoid false-positive results, negative controls included sterile water and *Bartonella*-negative fleas DNA were added as the template**s** in each run. DNA extracted from *B. elizabethae* culture (kindly provided by Pr. Zanzen, Hospital Hedi Chaker Sfax, Tunisia) was used as a positive control. To avoid cross-contamination, the extraction, amplification and gel electrophoresis were done in separate rooms. PCR products were visualized by ethidium bromide staining after electrophoresis in a 1.5% agarose gel (Gellyphor, EuroClone, Milan, Italy) and their size was estimated by comparing them with Gene RulerTM 100-bp DNA Ladder (MBI Fermentas, Vilnius, Lithuania) as a molecular marker. PCR of the 16S-23S rRNA Intergenic Spacer Regions (ITS) used the primers ITS2F (5′-GGG GCC GTA GCT CAG CTG-3′) and ITS2R (5′-TGA ATA TAT CTT CTC TTC ACA ATT TC-3′) [16]. For the ITS region, the PCR cycling conditions were performed as follows: 15 min at 94 °C for an initial denaturation followed by 35 cycles of 1 min at 94 °C, 35 s at 60 °C, and 30 s at 72 °C, with a final extension of 7 min at 72 °C. PCR products were visualized by ethidium bromide staining after electrophoresis in a 3% agarose gel (Gellyphor, EuroClone, Milan, Italy) and their size was estimated by comparing them with Gene RulerTM 50-bp DNA Ladder (Bio Basic Canada Inc., Markham, Canada). Gels were photographed using Gel Doc 2000 (Bio-Rad, Hercules, CA, USA).

All flea pools that had tested positive for the *gltA Bartonella* gene were confirmed by sequencing, targeting the two loci (*gltA*, ITS). Some PCR products were sequenced using the same oligonucleotides as for PCR to confirm the PCR results and to characterize the *Bartonella* species: PCR products were purified using the ExoSAP cleanup procedure (Amersham Biosciences, Piscataway, USA). All nucleotide sequences were obtained using the Big Dye Terminator v.3.1 Cycle Sequencing Kit (Applied Biosystems, Foster City, USA) and the 3130 automated sequencer (Applied Biosystems, Foster City, USA). Sequences were determined on both forward and reverse strands to maximize data accuracy for the two used loci. The *gltA* and ITS sequences identified in this study were submitted to the GenBank database (see Availability of data and materials). Data sequences were compared and analyzed using the BLAST program (http://www.ncbi.nlm.nih.gov/BLAST).

The CLUSTAL W algorithm was used for sequence alignments and MEGA 6.06 software for phylogenetic analyses. Sequences of the *gltA* gene were used to construct a phylogenetic tree using the neighbor-joining method.

### Statistical analysis

Estimated infection rates (EIR) of all analyzed pools and of each flea species with corresponding 95% confidence intervals (CI) were calculated, based on equal pool sizes (2 fleas/tube), using the binGroup package [17] in the software R (R Core Team 2015).

The Chi-square test (EpiInfo 6.04) was used to compare the *Bartonella* infection rate of flea pools by species, animal host and bioclimatic area. The observed differences were considered significant when the resulting *P-*value was <0.05.

## Results

### Collection and identification of fleas

A total of 2178 fleas (67% females, 33% males) were collected from 5 cats (*n* = 51), 27 dogs (*n* = 664), 34 sheep (*n* = 341) and 41 goats (*n* = 1122) from all sites in Tunisia where the investigation was carried out. The largest number of fleas was removed from goats*. Ctenocephalides felis* was found on all examined animals (cats, dogs, sheep and goats) and was the dominant species of those collected (83%; *n* = 1803), followed by *Ctenocephalides canis* (12%; *n* = 266) and *Pulex irritans* (5%; *n =* 109) (Table 1).

**Table 1:** The number of the identified flea species and *Bartonella* infection rate per flea pool by host species (*gltA*)

Flea species	Cat	Dog	Sheep	Goat	Total
*C. felis*	51	289	341	1122	1803
*C. canis*	0	266	0	0	266
*P. irritans*	0	109	0	0	109
Total	51	664	341	1122	2178
*Bartonella* infection rate per pool in fleas % (X/Y)	33.3 (8/24)	32.7 (97/296)	5.8 (10/170)	1.6 (6/376)	14 (121/866)

*Abbreviations*: *X* number of pools positive for *Bartonella* DNA, *Y* number of tested flea pools

A predominance of females was observed in the collected *C. felis* (1249 females, 554 males) and *C. canis* (158 females, 108 males), whereas for *P. irritans* males outnumbered females (60 males, 49 females).

Cats, sheep and goats were infested exclusively with *C. felis*, while dogs were infested with *C. felis*, *C. canis* and *P. irritans* (Table 1).

### PCR detection of *Bartonella* in collected fleas

Among the 2178 collected fleas, 1732 specimens pooled in 866 tubes (714 *C. felis* pools, 118 *C. canis* pools and 34 *P. irritans* pools) were screened for the presence of *Bartonella* DNA, which was detected in 14% (121/866) of the tested flea pools based on the *gltA* gene (EIR per 2 specimens: 0.072, 95% CI: 0.060–0.086); details are summarized in Table 2. The overall *Bartonella* infection rates per pool of *C. canis* and *P. irritans* were 55% (65/118) and 23.5% (8/34), respectively. For *C. felis*, only 6.7% (48/714) of the flea pool samples tested positive for *Bartonella* DNA (Table 2). The differences in the *Bartonella* infection rates per pool among the three flea species were statistically significant (*χ* ^2^ = 199.7; *df* = 2; *P* < 0.0001). The largest number of infected *C. felis* was removed from cats (33.3%; 8/24) and dogs (16.6%; 24/144). However, 5.8% (10/170) and 1.6% (6/376), of infected *C. felis* were collected from sheep and goats, respectively. The differences in numbers of infected *C. felis* observed by animal host were statistically significant (*χ* ^2^ = 65.7; *df* = 3; *P* < 0.0001). The *Bartonella* infection rate per pool of all identified flea species was analyzed by the animal host: The rates in the flea pools collected from cats (33.3%) and dogs (32.7%) was higher than in those collected from sheep (5.8%) and goats (1.6%) (Table 1). These differences were statistically significant (*χ* ^2^ = 151.6; *df* = 3; *P* < 0.0001). In this study we found that fleas (*C. felis*, *C. canis* and *P. irritans*) infected with *Bartonella* were collected from 11 of the 22 sites in the different bioclimatic areas (Fig. 1), where the *Bartonella* infection rates of flea pools varied significantly. The highest infection rate was observed in the humid zone (28%; 56/200), followed by the semi-arid (21.6%; 54/249), sub-humid (7%; 4/57) and arid zones (2%; 7/321). No *Bartonella* DNA was found in fleas collected in the Saharan areas (0%; 0/39) (*χ* ^2^ = 67.13; *df* = 4; *P* < 0.0001).

**Table 2:** The estimated infection rate of *Bartonella* over all analyzed pools and per each flea species with corresponding 95% confidence intervals (CI)

Flea species	Number of specimens	Pools tested	Positive pools	Estimated infection rate	95% CI	Infection rate per pool (%)
*C. felis*	1428	714	48	0.0342	0.0253–0.0450	6.7
*C. canis*	236	118	65	0.3298	0.2628–0.4021	55
*P. irritans*	68	34	8	0.1255	0.0552–0.2330	23.5
Total	1732	866	121	0.0724	0.0604–0.0860	14

### Sequence analysis

Only 81 out of 121 amplicons, obtained by targeting the *gltA* gene, were sequenced, while the remaining showed a weak positive signal on the electrophoresis gel. Sequencing made it possible to identify three zoonotic *Bartonella* species, *B. henselae*, *B. elizabethae* and *B. clarridgeiae*, as well as uncharacterized *Bartonella* genotypes. The number of sequencing samples and the distribution of *Bartonella* species detected in this study by bioclimatic zone, site, flea species, and animal host are summarized in Additional file 1: Table S1 and Table 3. Based on the *gltA* gene, a *Bartonella* sp. with an identity score between 98 and 100% with *B. henselae* isolated from human blood in France (GenBank: HG969191) was detected in 50 flea pool samples. A *Bartonella* sp. with 100% identity with *B. elizabethae* detected in dog ticks in Taiwan [18] (GenBank: GU056193), was detected in 8 flea pools. In addition, a *Bartonella* sp. with a 99% identity score with *B. clarridgeiae,* described in Switzerland [19] (GenBank: FN645454), was detected in 4 flea pools. A *Bartonella* sp. with an identity score varying between 97 and 100% with uncultured *Bartonella* sp. clone B224RnF isolated from rodents [20] (GenBank: KC763936), was detected in 8 flea pools. In addition, a *Bartonella* sp. with an identity score varying between 99 and 100% with *Bartonella* sp. BR10 detected in dog ticks in Taiwan [18] (GenBank: GU056200), was detected in 11 flea pools (Additional file 1: Table S1).

**Table 3:** Number of *Bartonella* species, detected using the *gltA* gene, in the identified flea species collected in Tunisia

Flea species	*B. henselae*	Uncharacterized *Bartonella* genotypes	*B. elizabethae*	*B. clarridgeiae*
*C. felis*	26	1	8	4
*C. canis*	24	10	0	0
*P. irritans*	0	8	0	0

By sequencing the ITS region of 46 amplicons out of 81, we were able to confirm the identification of the three zoonotic *Bartonella* species (*B. henselae*, *B. elizabethae* and *B. clarridgeiae*) detected by the *gltA* gene, with an identity score between 99 and 100% with the published sequences in GenBank (HG969191, L35103 and FN645454, respectively). In addition, a *Bartonella* sp. with an identity score of 100% with *Bartonella* sp. Lao/Nh2 isolated from rodents (GenBank: EU714977) [21], was detected in 8 flea pools that were identified as Uncultured *Bartonella* sp. B224RnF clones TUN by *gltA* gene. Targeting the ITS region, we were unable to amplify and sequence the pools revealed positive for Uncultured *Bartonella* sp. BR 10 TUN clones by the *gltA* gene (Additional file 1: Table S1).

**Fig. 2:**
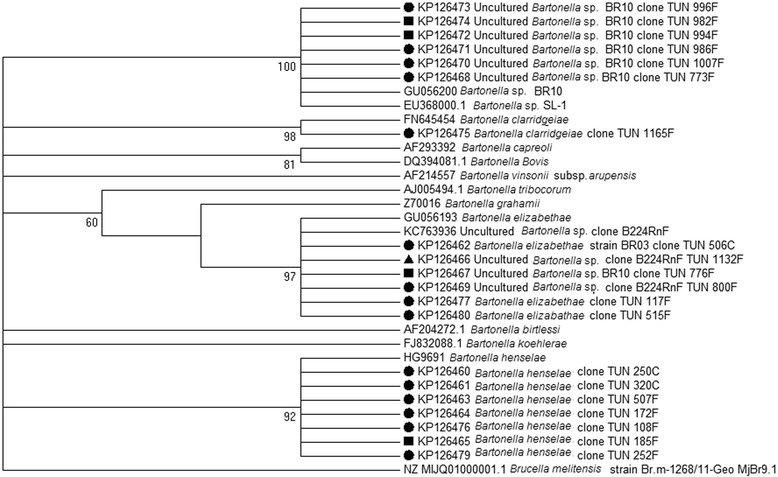
Phylogenetic tree based on citrate synthase (*gltA)* gene of *Bartonella* spp. using the MEGA 5.02 software. The tree was obtained using the neighbor-joining method. Numbers at the nodes are proportions of 1000 bootstrap resampling that support the topology. *Brucella melitensis* strain Br.m-1268/11-Geo MjBr9.1 was used as the outgroup to root the tree. TUN (Tunisia) sequences detected in this work have been deposited in GenBank under the accession numbers KP126460 – KP126480. ●: *Ctenocephalides felis*; ■: *Ctenocephalides canis*; *▲: Pulex irritans*

A *gltA* phylogenetic tree made it possible to visualize the arrangement of the *Bartonella* species identified in our study as compared to those identified in other studies. It showed that Tunisian sequences were clustered in 4 different groups (Fig. 2).

## Discussion

Fleas are blood-sucking ectoparasites that are important vectors of pathogens including the bacteria of the *Bartonella* genus. In our study, 2178 fleas were removed from cats, dogs, sheep and goats from rural and urban sites in Tunisia’s five bioclimatic zones. These animals spend most of their time outdoors exclusively. We observed a severe flea infestation in goats on several farms. Such highly infested animals can suffer from anemia and die as a result of untreated flea infestations, and create significant financial losses for their owners [22]. The collected fleas were identified as *C. felis*, *C. canis* and *P. irritans.* The cat flea, *C. felis* (83% of all collected fleas), an ubiquist ectoparasite, was found on all investigated animal species in all regions. The same predominance has been observed in Germany, where Beck et al. [23] identified 81% of the fleas removed from dogs and cats as *C. felis*, followed by *C. canis* and *P. irritans*. A survey conducted in Iran also showed *C. felis* to be the most frequent species infested dogs (42%), followed by *P. irritans* (26.5%) and *C. canis* (16.5%) [24]. The same observations have been reported elsewhere in North Africa, in Egypt [25], Algeria [26], Libya [22] and Morocco [27]. Such data show the adaptation of cat fleas to other animal species. By contrast, *C. canis* collected from dogs was most prevalent in Ireland [28] and New Zealand [29].

*Ctenocephalides canis* and *P. irritans* were found only on dogs in northern Tunisia (Tunis, Béni Khiar, Jouza); 40.7% (11/27) had a mixed infestation of *C. felis*, *C. canis* and *P. irritans* (Table 1). Our finding is unsurprising, since *P. irritans*, which is normally associated with humans, has also been detected on dogs in Libya [22], Morocco [27], France [30] and Iran [24].

Our study showed that *Bartonella* DNA was detected in 14% of flea pools analyzed by the *gltA* gene. This rate is lower than those in studies from the UK (17%; flea pools) [31] and France (26.2%; single specimens) [32], but higher than those in studies from the USA (11.3%; flea pools) [33], Israel (7.8%; flea pools) [34], Hungary (4%; flea pools) [35] and in countries neighboring Tunisia such as Morocco (4%; single specimens) [27] and Algeria (9.33%; single specimens). Our study showed variations in the *Bartonella* infection rates of flea pools in the different bioclimatic zones. The rates were highest in the humid area. No *Bartonella* infection was reported in the Saharan zone, confirming the results of a recent study undertaken in Tunisia showing that *Bartonella* seropositivity rates in dogs were highest in the humid zone [13].

The variation of *Bartonella* infection rates in countries, bioclimatic zones, and sites could be attributed to the detection methodologies used (culture or molecular approach), or to such bioclimatic parameters as temperature and humidity, the principal factors influencing flea survival, development, reproduction, and the abundance of *Bartonella* reservoirs. Fleas must be therefore carefully studied to better understand the relationships among climatic factors, *Bartonella* hosts and vectors [24, 36, 37].

The *Bartonella* infection rates in the pools also vary with flea species: 55% for *C. canis*, 23.5% for *P. irritans* and 7% for *C. felis*. In Japan, all tested (single specimens) *C. canis* were infected with *Bartonella* compared to 33% in *C. felis* [38]. In Germany, the *Bartonella* infection rates of *C. felis* and *C. canis* were 2.4% and 0.4%, respectively [32].

The positive PCR products were sequenced and analyzed in this study; thus, we were able to report for the first time in Tunisia that fleas harbor three known *Bartonella* species, *B. elizabethae*, *B. henselae* and *B. clarridgeiae*, as well as uncharacterized *Bartonella* genotypes. The first three species are zoonotic and were also reported in fleas in neighboring countries, where *B. clarridgeiae* and *B. henselae* were identified in *C. felis* collected from cats, goats and sheep in Morocco [27]. In Algeria, *B. henselae*, *B. clarridgeiae* and *B. vinsonii* *berkhoffii* were detected in *C. felis* and *Xenopsylla cheopis* collected from cats and dogs [26] whereas *B. elizabethae*, *B.tribocorum* and *B. clarridgeiae* were detected in fleas collected from hedgehogs, rats, and mice [39]. Based on the *gltA* gene and the ITS, we detected genotypes of uncultured *Bartonella* sp. (*Bartonella* sp. BR10 TUN, Uncultured *Bartonella* sp. clone B224RnF TUN and *Bartonella* sp. Lao/Nh2 TUN). *Bartonella* sp. BR10 TUN was detected in *C. felis* and *C. canis* collected from dogs. This *Bartonella* species was also detected in fleas (*Pulex simulans*) and in brown dog ticks (*Rhipicephalus sanguinus*) collected from dogs in Taiwan and Costa Rica (Central America), respectively [18, 40]. In our study, the DNA of uncultured *Bartonella* sp. clone B224RnF TUN (*gltA*) and uncultured *Bartonella* sp. Lao/Nh2 TUN (ITS) described in *P. irritans* collected from dogs were found in rodents in the USA and in Southeast Asia [20, 21].

The *gltA* phylogenetic tree shows that sequences of uncultured *Bartonella* sp. clone B224RnF TUN group in the same cluster with *B. elizabethae* TUN and very closely to *B. tribocorum* and *B. grahamii*, which are commonly detected in rodents and micromammals (Fig. 2) [20, 21]. This suggested that *Bartonella* sp. clone B224RnF TUN probably belongs to the rodent-borne pathogens. However, additional molecular markers are needed to identify the uncharacterized *Bartonella* genotypes. *Bartonella* species detected in fleas show that *B. henselae* (61.7%) was most frequent followed by uncharacterized *Bartonella* genotypes (23.4%), *B. elizabethae* (9.8%) and *B. clarridgeiae* (4.9%) (Additional file 1: Table S1, Table 3). We report that *C. felis* harbor different *Bartonella* species (*B. henselae*, *B. elizabethae*, *B. clarridgeiae* and uncharacterized *Bartonella* genotypes) while in *C. canis* we detected only *B. henseale* and uncharacterized *Bartonella* genotypes. Only uncharacterized *Bartonella* genotypes were detected in *P. irritans* (Table 3). The high frequency of *B. henselae* in our survey is unsurprising, given that it is considered to be the most common *Bartonella* species in infected fleas, mainly in *C. felis* [41]. In Morocco, *B. henselae*, the main agent of CSD, was also detected in *C. felis* collected from goats and sheep [27]. This species can be characterized by atypical symptoms and complications in 5–15% of CSD patients who may have encephalitis, retinitis, osteitis, atypical pneumonitis, neurological syndromes, and prolonged fever [41]. *Bartonella henselae* can multiply in the digestive system of the cat flea and survive for several days in flea feces [8].

In Tunisia, *Bartonella* endocarditis seems to be common. Indeed, 9.8% of infective endocarditis was caused essentially by *B. quintana*, while 2.5% were caused by *B. henselae* [9, 10]. The latest bacterium has also been involved in intraocular inflammation in Tunisian patients with CSD [9]. Humans are the principal, albeit not the sole, reservoir hosts for *B. quintana*, which was isolated from other hosts, including a cynomolgus monkey in Ireland [42] and dogs in New Zealand [43]. Endocarditis caused by *B. quintana*, which is a louse-borne pathogen, was associated with homelessness [44]. Recently, *B. quintana* DNA was detected in cat fleas, which means that this flea species may be an unrecognized vector for the transmission among cats of *B. quintana* [34]. It was also detected in the dental pulp of a cat in France [45] and was isolated in a cat and its owner in the USA [46].

*Bartonella* sp. was first reported in Tunisia in 49% of fat rats, *Psammomys obesus*, captured in the arid zone [46]. A molecular and serological study proved the presence of “*Candidatus* B. merieuxii”, *B. vinsonii*, *B. henselae*, *B. clarridgeiae* and *B. bovis*, correlating with tick and/or flea infestations of examined dogs [12, 13]. Humans and dogs are accidental hosts of *B. henselae*, whereas cats are their natural reservoir [47]. *Bartonella vinsonii berkhoffii*, *B. henselae* and *B. rochalimae* are the main species known to infect canids [47]. Recently, many other *Bartonella* species, including *B. elizabethae,* have been detected in domestic dogs [48], confirming our finding insofar as fleas collected from dogs were positive for *B. hensale* and *B. elizabethae*. We also reported that small ruminants were infested with *C. felis* that were positive for *B. elizabethae*, a rodent-associated zoonotic *Bartonella* species. It is likely that dogs, sheep and goats were infected with *B. elizabethae*, or that the fleas had been exposed to infected rodents. *B. elizabethae* DNA was previously detected in dog blood in the USA [49] and in fleas collected from dogs in Israel [32]. To the best of our knowledge, this is the first report of *B. elizabethae* in fleas collected from sheep and goats. Infections caused by *B. elizabethae* in humans have been associated with endocarditis and neuroretinitis [50, 51].

Our results revealed that fleas infesting domestic cats were positive for *B. clarridgeiae* DNA. This species was first isolated in the USA, where a patient displayed worrying clinical signs six months after adopted an infested kitten [52]. Several studies report that *C. felis* could be a potential vector for *B. clarridgeiae* [53, 16], which was isolated from cats and dogs. Other surveys report that dogs living in rural areas and exposed to ticks and fleas are at greater risk of being infected with *Bartonella* spp.[54, 55].

## Conclusions

Our study shows the presence of three flea species infesting domestic animals in Tunisia; *Ctenocephalides felis* was predominant in comparison to *C. canis* and *P. irritans* fleas. To our knowledge, this study has shown for the first time in Tunisia, that fleas carry three zoonotic *Bartonella* species, *B. elizabethae*, *B. henselae* and *B. clarridgeiae*, as well as uncharacterized *Bartonella* genotypes**.** Our results indicate that the dog flea, *C. canis*, should be considered to be the principal potential vector of *Bartonella*. The highest *Bartonella* infection rate of flea pools was recorded in the humid area of northern Tunisia. These data may open up prospects for further studies of the effect of bioclimatic factors on the biology of fleas and their infection by *Bartonella.* Extensive molecular studies are needed to identify the uncharacterized *Bartonella* genotypes. Given the high rate of *Bartonella* infection in fleas in Tunisia, medical practitioners, pet owners and farmers should be informed about the vectorial role of fleas in transmitting *Bartonella*.

## Additional file

Additional file 1: Table S1. Distribution of *Bartonella* species by bioclimatic zones, site, flea species and animal host and partial sequencing analysis of *gltA* gene and the ITS region. (PDF 23 kb)

## Abbreviations

CI: Confidence interval
CSD: Cat-scratch disease
EIR: Estimated infection rate
*gltA*: citrate synthase gene
ITS: 16S-23S rRNA intergenic spacer region
MLVA: Multiple locus variable number tandem repeats analysis
MST: Multilocus sequence typing
NCBI: National Center for Biotechnology Information
RFLP: Restriction fragment length polymorphism
