# Supplementary material for: First molecular detection and characterization of zoonotic Bartonella species in fleas infesting domestic animals in Tunisia
Source: Parasit Vectors. 2017 Sep 19;10:436. doi: 10.1186/s13071-017-2372-5 (PMC5606017; doi:10.1186/s13071-017-2372-5)
Supplement: Additional file 1: Table S1. — Distribution of Bartonella species by bioclimatic zones, site, flea species and animal host and partial sequencing analysis of gltA gene and the ITS region. (PDF 23 kb) [file 13071_2017_2372_MOESM1_ESM.pdf]

Additional file 1: Supplemental Table S1. Distribution of *Bartonella* species by bioclimatic zones, site, flea species and animal host and partial sequencing analysis of *gltA* gene and the ITS region.

**Bioclimatic zones**	**Sites**	**hosts**	**Flea species**	***gltA***	**ITS**	**X/Y**
				***Bartonella* species (n)**		
**Humid**	Jouza	Dogs	*C. canis*	Uncultured *Bartonella* sp. BR10 clones TUN (7)<br>*B. henselae* (6)	*B. henselae* (4)	
			C. felis	Uncultured *Bartonella* sp. BR10 clones TUN (1)<br>*B. henselae* (17)	*B. henselae* (10)	31/56
**Sub-humid**	Ichkeul	Sheep	*C. felis*	*B. elizabethae* (4)	*B. elizabethae* (3)	4/4
**Semi-arid**	El Mabtouh	Sheep	*C. felis*	*B. henselae* (4)	*B. henselae* (1)	
	Oudhna	Sheep	*C. felis*	*B. henselae* (1)	*B. henselae* (1)	
	El Azima	Dogs	*C. canis*	Uncultured *Bartonella* sp. BR10 clones TUN (3)		

	El Hessiane	Dogs	*C. canis*	*B. henselae* (18)	*B. henselae* (10)	40/54
	Bab Saadoun	Cats	*C. felis*	*B. clarridgeiae* (4)	*B. clarridgeiae* (4)	
	Béni Khiar	Dogs	*P. irritans*	Uncultured *Bartonella* sp. B224RnF clones TUN (8)	Uncultured *Bartonella* sp. Lao/Nh2 clone TUN (8)	
	Maloulech	Dogs	*C. felis*	*B. elizabethae* (2)	*B. elizabethae* (1)	
**Arid**	Sfax Sud	Goats	*C. felis*	*B. henselae* (4)	*B. henselae* (2)	6/7
	Zarzis	Goats	*C. felis*	*B. elizabethae* (2)	*B. elizabethae* (2)	
**Saharan**	Tataouine	Goats	*C. felis*	none	none	0
	Kbeli	Goats	*C. felis*			

X: number of the sequenced positive flea pools (ITS and *gltA* gene).

Y: Number of the positive flea pools by PCR (ITS and *gltA* gene).
